# Clinical profiles and treatment outcomes of outpatients with interstitial lung disease and mechanic’s hands: A retrospective and observational cohort

**DOI:** 10.1097/MD.0000000000038642

**Published:** 2024-06-28

**Authors:** Gustavo Frazatto Medeiros de Miranda, Maria Raquel Soares, Alexandre Wagner Silva de Souza, Luis Eduardo Coelho Andrade, Carlos Alberto de Castro Pereira

**Affiliations:** a Department of Medicine, Discipline of Pulmonology, Federal University of Sao Paulo, Sao Paulo, Brazil; b Department of Medicine, Discipline of Rheumatology, Federal University of Sao Paulo, Sao Paulo, Brazil.

**Keywords:** antisynthetase syndrome, idiopathic inflammatory myopathies, interstitial lung disease, mechanic’s hands

## Abstract

Idiopathic inflammatory myopathies, especially antisynthetase syndrome, often appear outside of the muscles as interstitial lung disease (ILD). Another typical finding is the presence of mechanic’s hands. The aim of the present study was to describe the clinical, functional, tomographic, and serological data of patients with ILD and mechanic’s hands and their response to treatment and survival rates. This is a retrospective study of ILD with concurrent myopathy. Among the 119 patients initially selected, 51 had mechanic’s hands. All the patients were screened for anti-Jo-1 antibodies. An expanded panel of myopathy autoantibodies was also performed in 27 individuals. Of the 51 patients, 35 had 1 or more antibodies. The most common were anti-Jo-1, anti-PL-7, and anti-PL-12, while of the associated antibodies, anti-Ro52 was present in 70% of the 27 tested individuals. A significant response to treatment was characterized by an increase in predicted forced vital capacity (FVC) of at least 5% in the last evaluation done after 6 to 24 months of treatment. A decrease in predicted FVC of at least 5%, the need for oxygen therapy, or death were all considered treatment failures. All patients were treated with corticosteroids, and 71% with mycophenolate. After 24 months, 18 patients had an increase in FVC, 11 had a decrease, and 22 remained stable. After a median follow-up of 58 months, 48 patients remained alive and three died. Patients with honeycombing on high-resolution chest tomography (log-rank = 34.65; *P* < .001) and a decrease in FVC ≥5% (log-rank = 18.28, *P* < .001) had a poorer survival rate. Patients with ILD and mechanic’s hands respond well to immunosuppressive treatment.

## 1. Introduction

Idiopathic inflammatory myopathies (IIM) often develop outside the muscles as interstitial lung disease (ILD), especially in antisynthetase syndrome (ASS).^[1]^ Many of these cases may have no muscle manifestation but have features of ILD. A set of diagnostic criteria for ASS have been proposed, with the presence of anti-aminoacyl-tRNA synthetase antibodies, ILD, and polymyositis/dermatomyositis as major criteria and mechanic’s hands, arthritis, and Raynaud phenomenon as minor criteria.^[2,3]^ ILD and any of the common clinical features of ASS should be tested for antisynthetase autoantibodies, because often have incomplete syndrome at the initial presentation.^[4–6]^

In IIM, autoantibodies are classified as myositis-associated autoantibodies (MAAs) and myositis-specific autoantibodies (MSAs).^[7,8]^ The MAAs are not specific to polymyositis and dermatomyositis and can be found in a variety of autoimmune diseases.

One of the common clinical symptoms of ASS is mechanic’s hands, a condition characterized by scaly, hyperkeratotic, fissured, hyperpigmented eruptions typically on the ulnar surface of the thumb and the radial aspect of the fingers, most notable on the index and middle fingers.^[9]^ In the first published series of patients with ASS (n = 47), mechanic’s hands were noted in 71% of the cases.^[10]^ In another study, patients with mechanic’s hands had an odds ratio of 3.28 (95% confidence interval, 1.37–7.88) for ILD when compared to those without mechanic’s hands (*P* = .01).^[11]^

The importance of studying the phenotype of ILD with mechanic’s hands is to identify common clinical characteristics that can be clinically identified, even in the unavailability of MSAs or MAAs, with the aim of describing the clinical, functional, tomographic and serological tests and describe the response to treatment and factors related to survival, hypothesizing worse survival in those with greater functional decline.

## 2. Material and methods

### 2.1. Study population and design

This was a retrospective study of consecutive cases of ILD with concurrent IIM follow-up between February 2009 and December 2020 in the Interstitial Pulmonary Diseases Center at the Federal University of São Paulo. Medical appointments were accomplished every 4 months or early in some cases, such as medication adjustment, pulse therapy, and checking results. The data were collected from April 2017 and finished in October 2021. ILD was characterized by high-resolution chest tomography (HRCT) and IIM by the presence of 1 or more of the following: mechanic’s hands, increased muscle enzymes (above 2.5 times the upper normal limit), and specific serological autoantibodies.^[12–14]^ A multidisciplinary team made up of skilled pulmonologists, radiologists, rheumatologists, and pathologists evaluated all cases. Pulmonary function tests were performed in accordance with the recommendations suggested by the American Thoracic Society (ATS), European Respiratory Society (ERS), and Brazilian Society of Pulmonology and Phthisiology (*Sociedade Brasileira de Pneumologia e Tisiologia*),^[15,16]^ and lung function was serially measured every 3 to 6 months, depending on severity. Oxygen saturation at rest and its variation were obtained after performing the 4-minute step test.^[17]^

Individuals over the age of 18 years, with ILD who underwent functional and tomographic evaluations within 3 months of the initial visit and who had mechanic’s hands were the included in the study and followed by clinical consultations, with data saved in a standardized electronic medical record. The exclusion criteria were the absence of functional or imaging tests available within 3 months of diagnosis, symptoms for more than 6 years, or overlap with confounding diseases such as systemic sclerosis.

### 2.2. Evaluation

Tomographic patterns were categorized into 3 groups: reticular with honeycombing; ground glass or consolidation with fibrosis; and ground glass or consolidation without fibrosis. Surgical lung biopsy was performed in unclear cases, such as the absence of serum autoantibodies or the need for differential diagnosis. Following the pathological criteria recommended by the ATS/ERS, a qualified pathologist performed the analysis.^[14]^ Patients without CT scan, lung function, follow-up, or incomplete data were excluded. Pulmonary function tests were performed in accordance with the recommendations suggested by the ATS/ERS and with predicted values for the Brazilian population.^[15,16]^

HEp-2 cell indirect immunofluorescence (HEp-2 IFA) and anti-Jo-1 antibodies were used to detect autoantibodies in all patients. An expanded panel for myopathy antibodies was also performed in 27 patients using the 16-antibody line blot assay DL 1530-1601-4 G from EUROIMMUN Medical Laboratory Diagnostics AG (Lubeck, Germany). The reading was performed according to the manufacturer’s recommendations by a rheumatologist specializing in autoantibody testing (LECA). Only strong or very strong reactors were considered positive. All the patients were screened for anti-Jo-1 antibodies.

### 2.3. Statistical analysis

Data were expressed as absolute frequency and percentage, mean and standard deviation, or median and interquartile range, whenever appropriate. Outlier database values were reviewed. A significant response to treatment was characterized by an increase in predicted forced vital capacity (FVC) of at least 5% in the last evaluation done after 6 to 24 months of treatment. A decrease in predicted FVC of at least 5%, the need for oxygen therapy, or death were all considered treatment failures. Missing data associated with rheumatological complaints, such as antibody measurement, were reported as absolute frequency.

Survival time was assessed from the date of the first consultation until death or loss to follow-up. The last evaluation was obtained from telephone interviews or medical records on December 1, 2020. Mortality from all causes was calculated using the Kaplan–Meier method. Statistical significance was set at *P*-value ≤ .05. Survival was also compared between the stable subgroup and those with improved FVC using the Kaplan–Meier method. Calculations were performed using SPSS v-23 statistical package (2015, IBM^®^ SPSS^®^ Statistics, Armonk, NY).

The study protocol was approved by the Research Ethics Committee of the Federal University of São Paulo under number 0857/2019.

## 3. Results

**Figure 1.**
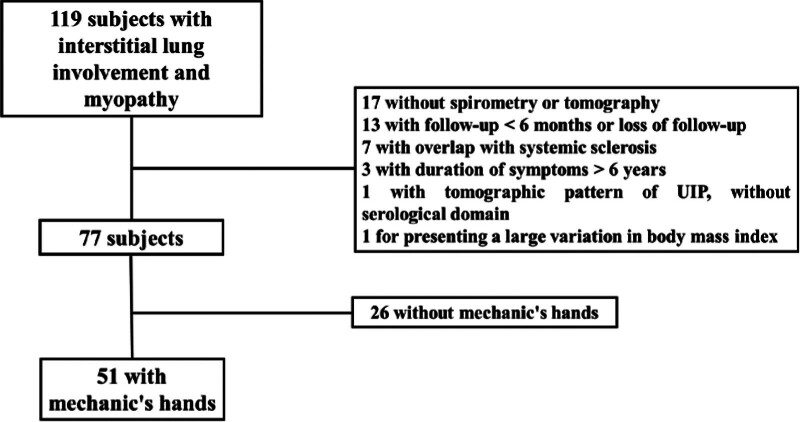
Stratification of patients for analysis.

**Figure 2.**
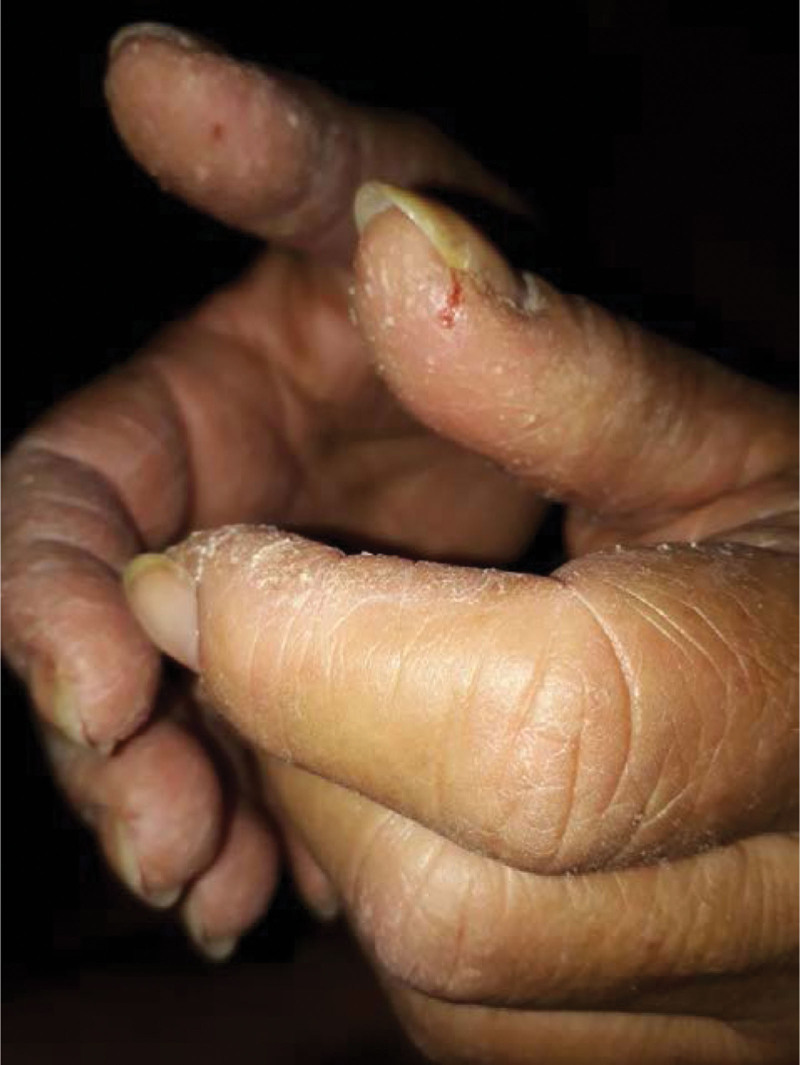
Photograph of patients’ hands with the presence of mechanic’s hands.

Among the 119 patients with findings of ILD and myopathy, 42 were excluded because they did not meet the eligibility criteria, and 26 did not have mechanic’s hands, leaving 51 remaining patients for analysis (Fig. 1). The presence of mechanic’s hands was characterized by visual inspection (Fig. 2). The baseline characteristics and clinical data of the patients are shown in Table 1. Most patients were females, with a mean age of 51 years, and the median duration of symptoms prior to attending the center was 11 months. The median follow-up was 5 years.

**Table 1:** General characteristics of patients.

General features	
Age, yr x̄ ± SD	50.6 ± 9.2
Female n (%)	32 (63%)
Time of symptoms Md, [IQR] months	11 [5–36]
Follow-up time Md, [IQR] months	58 [26–115]
Smoking history n (%)	22 (44%)
mMRC 1/2/3/4	18/24/9/0
Baseline FVC (%) x̄ ± SD	62.6 ± 17.2
Rest SpO_2_ to exertion (%) x̄ ± SD	95 ± 2.6/87.7 ± 6.5
Velcro crackles n (%)	40 (78.4%)
Gastroesophageal reflux symptoms	41 (80%)
Tomographic pattern	
OP + NSIP without fibrosis	18 (35.3%)
NSIP with fibrosis	29 (56.8)
UIP	4 (7.8%)
Surgical lung biopsy	10 (19.6%)
Lymphoplasmacytic inflammation	3 (5.9%)
NSIP	2 (3.9%)
UIP	2 (3.9%)
OP	1 (1.9%)
NSIP + OP	1 (1.9%)
Diffuse alveolar damage	1 (1.9%)
Deep vein thrombosis	4 (7.8%)
Cancer	1 (1–9)

% = percentage, FVC = forced vital capacity, IQR = interquartile range, Md = median, mMRC = Modified Medical Research Council, n = number, NSIP = nonspecific interstitial pneumonia, OP = organizing pneumonia, SD = standard deviation, SpO_2_ = peripheral capillary oxygen saturation, UIP = usual interstitial pneumonia, x̄ = mean.

Dyspnea was mild to moderate; mean FVC was 63% of predicted and usually normal oxygen saturation at rest with a decrease in exercise by 4-minute step test.^[17]^ Velcro crackles were heard in 78% of the patients. HRCT indicated that fibrosis was present in 64% of patients. Eighty percent of the subjects reported gastroesophageal reflux symptoms. Surgical lung biopsy was performed in 10 patients, with nonspecific interstitial pneumonia identified in 2, organizing pneumonia in 1, and a combination of both in 1 case. Lymphoplasmacytic inflammation was observed in 3 patients, diffuse alveolar damage in 1, and usual interstitial pneumonia in 2. In addition, 4 individuals had deep vein thrombosis, and 1 also had a pulmonary embolism. Prostate cancer was diagnosed in one patient.

The main complaints of systemic autoimmune rheumatic diseases were myalgia (80%; n = 32/40), muscle weakness (73%; n = 33/45), and arthralgia (64%; n = 32/50). Elevated muscle enzymes (creatine kinase or aldolase) were present in 41% (n = 19/46) of patients, and Raynaud phenomenon in 33% (n = 16/48). Of the 51 patients, 35 had 1 or more of the associated antibodies. The most common antibodies for MSAs were anti-Jo1, anti-PL7, and anti-PL12. The most common antibodies for MAAs were anti-Ro52, which was found in 70% of the people tested (Table 2). Two patients were concomitantly positive for anti-Jo1 and anti-PL7. In the final analysis, they were considered positive for anti-PL7.

**Table 2:** Absolute frequency of MAAs and MSAs.

Absolute frequency of MSAs	n	%
Anti-Jo-1 (n = 51)	17	33.3
Anti-PL-7 (n = 27)	4	14.8
Anti-PL-12 (n = 27)	3	11.1
Anti-Jo-1 + anti-PL-7 (n = 27)	2	7.4
Anti-MDA5 (n = 27)	1	3.7
Anti-EJ (n = 27)	1	3.7
Anti-EJ + MDA5 (n = 27)	1	3.7
Anti-SRP (n = 27)	1	3.7
Absolute frequency of MSAs		
Anti-Ro52 (n = 27)	14	51.8
Anti-Ro52 isolated (n = 27)	3	11.1
Anti-Ro52 + anti-Ku (n = 27)	1	3.7
Anti-Ro52 + PM/Scl (n = 27)	1	3.7
PM/Scl (n = 27)	1	3.7

MAAs = myositis-associated autoantibodies, MSAs = myositis-specific autoantibodies.

An antinuclear antibody (ANA) titer of ≥1:320 was observed in 26 (51%) individuals. None had nucleolar or centromeric patterns, and none had an antitopoisomerase antibody (anti-Scl-70). Of those with an ANA titer of ≥1:320, 14 displayed cytoplasmic patterns. Conor criteria for ASS were met by 59% of the individuals, while Solomon criteria were met by 43%. Bohan and Peter criteria were fulfilled in 26% of the cases. All patients met the criteria for interstitial pneumonia with autoimmune findings. In respect of the functional evolution during the follow-up period, 35% (n = 18) improved, 43% (n = 22) remained stable, and 22% (n = 11) worsened (Table 3).

**Table 3:** Changes in FVC % predicted after 6–24 mo of follow-up.

Change in FVC	n	%
Improvement ≥5%	18	35.3
Stable	22	43.1
Decrease ≥5% predicted	11	21.6

FVC = forced vital capacity.

**Figure 3.**
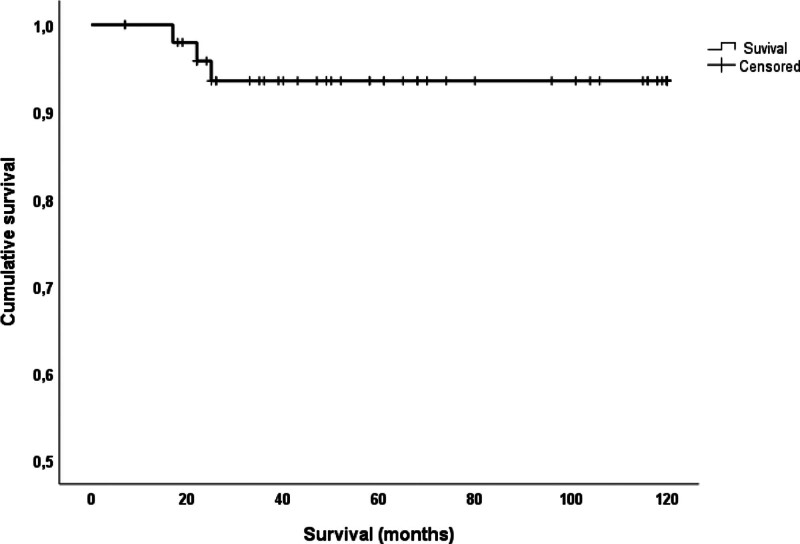
Global patient’s survival.

**Figure 4.**
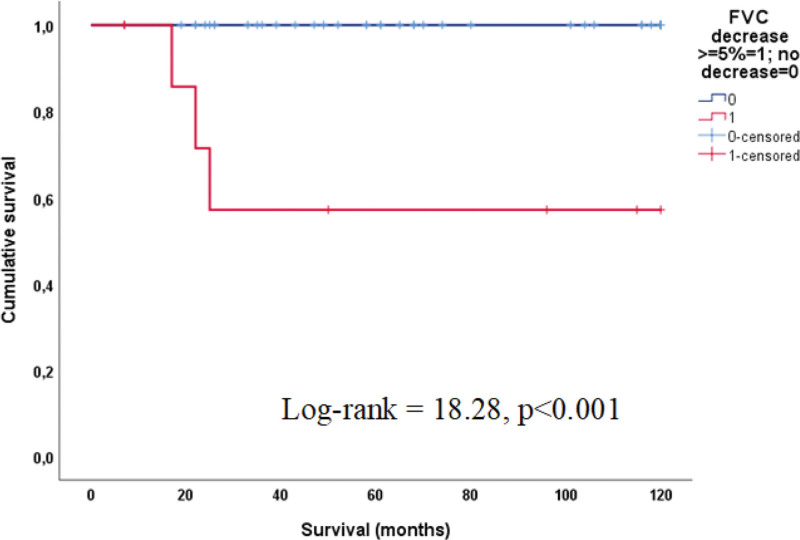
Survival of patients comparing cases with and without FVC decrease. FVC = forced vital capacity.

Table 4 shows data regarding the therapeutic modalities prescribed for the patients. Corticosteroids were administered to all patients, and mycophenolate was the most commonly used immunosuppressant. The most severe cases were treated with cyclophosphamide followed by mycophenolate or azathioprine. At least 1 relapse was observed in 14 patients (27%). Overall, survival was >90% at 5 years (Fig. 3). Death occurred in 3 patients (6%). Honeycombing was present in 4 individuals and was associated with worse survival (log-rank = 34.65; *P* < .001). Nine patients had a ≥5% decrease in predicted FVC values after 6 to 24 months of treatment. This was linked to a lower chance of survival (log-rank = 18.45; *P* < .001) (Fig. 4). In the comparative subanalysis between the group with clinical stability and improved FVC, there was no statistical difference in survival between these 2 groups, showing stability as a factor in better survival, as well as improved FVC.

**Table 4:** Treatment.

Treatment			n	%
Treatment time Md (IQR), mo				
	Corticosteroid time	38 [23–66]		
	Immunosuppressant time	36 [24–67]		
Medication class	Corticosteroid		51	100.0
	Mycophenolate		36	70.6
	Cyclophosphamide		31	60.8
	Azathioprine		26	50.1
	Cyclosporine		3	5.9
	Rituximab		1	2.0

IQR = interquartile range, Md = median.

## 4. Discussion

Our study shows that patients with ILD associated with the mechanic’s hands phenotype respond well to immunosuppressive treatment. Honeycombing on HRCT or a decrease in FVC ≥5% in this outpatient cohort were indicators of lower survival.

### 4.1. Mechanic’s hands

In the present study, 51 (43%) of 119 patients with ILD and findings of IIM had mechanic’s hands. In a meta-analysis of 3487 patients with ASS, the presence of mechanic’s hand was reported in 28% (95% confidence interval, 24%–33%).^[18,19]^ Stahl et al^[20]^ first described cutaneous lesions on the hands associated with myopathy in 1979. Mechanic’s hands are characterized by fissured, erythematous, and hyperkeratotic eruptions, resembling the type of occupational dermatosis that might be seen in a manual laborer. The lesions are classically on the lateral edges of the fingers and thumbs, sparing the palmar and dorsal aspects of the digits (Fig. 2).^[21]^ Occasionally, these lesions may occur in the feet, known as hiker’s feet.^[22,23]^ Mechanic’s hands are described in IIM but can overlap with other autoimmune rheumatic diseases, usually associated with the presence of anti-polymyositis/Scl, anti-MDA-5, anti-Mi2, anti-TIF1-gamma, and anti-U1-RNP antibodies.^[24–31]^ The pathogenesis of mechanic’s hands is still obscure, but persistent inflammation triggered by autoimmunity could stimulate uncontrolled keratinocyte proliferation via the tumor necrosis factor-α, interleukin (IL)-17, and interferon (IFN)-γ pathways.^[31]^ The actual diagnosis of mechanic’s hands is made by examining its clinical features, as histopathologic findings are nonspecific.^[32]^ Immunosuppressive treatment reduces hyperkeratosis and erythema, and the worsening of lesions may be related to disease relapse.^[33]^

There are several important points regarding the association between mechanic’s hands and ASS that should be considered: the combination of mechanic’s hands, myopathy, and ILD is not exclusive to ASS; mechanic’s hands can be present in cases without detectable MSAs; the presence of mechanic´s hands, even without satisfying the criteria for ASS, is a risk factor for ILD, a potentially severe and recalcitrant condition if not detected early.^[9,25–28,34–36]^

### 4.2. ILD and pulmonary symptoms

A meta-analysis showed that 70% of patients with ASS have ILD, mainly non-anti-Jo1 anti-aminoacyl tRNA synthetase (anti-ARS).^[19]^ ILD is the first symptom of myositis in about 40% of ASS, and the diagnosis of myopathy can be delayed because there are no musculoskeletal symptoms or an increase in muscle enzymes.^[19,25,37–39]^ Thus, diagnosis by clinicians and pulmonologists is crucial, as the rheumatological criteria for IIM may be initially absent or may not meet the obvious criteria.^[40,41]^

The most common histopathological patterns found in lung biopsy were organizing pneumonia, nonspecific interstitial pneumonia, and their associations.^[42,43]^ Interestingly, the presence of lymphoid aggregates was also identified as an associated finding. The indications for lung biopsy were diverse, including the presence of a possible alternative diagnosis and the absence of indicative antibodies.^[44,45]^

### 4.3. Autoantibodies and rheumatological manifestations

In our cohort, the most frequently found autoantibody was anti-Jo-1, as has been described in a systematic review and meta-analysis.^[7]^ However, in a study that used an expanded panel of autoantibodies, a higher frequency of non-Jo-1 antibodies was found (only 36% anti-Jo-1).^[5]^ In our cohort, there were 2 patients with 2 anti-ARS simultaneously (both with anti-Jo-1 and anti-PL-7). Finding more than 1 MSA is not common, but can be found.^[5,46]^ Interestingly, 4 of our subjects did not show any MSA or MAA. This could be related to fluctuating serum levels with transiently negative tests. Therefore, a negative autoantibody panel cannot exclude at diagnosis of IIM.^[47]^

In the present study, 2 cases had anti-MDA-5 and survived. It is well documented in the literature that the onset of clinical symptoms with anti-MDA-5 can be catastrophic, leading to diffuse alveolar damage, respiratory failure, and death.^[25,48–50]^ In an outpatient setting, it is possible to find patients with anti-MDA-5 with a chronic course similar to other myopathies or less severe cases, as shown in the present series.^[39]^

Autoantibodies detected in the HEp-2 IFA test were present in 51% of patients with titers ≥1:320, reinforcing that a negative HEp-2 IFA test does not exclude myopathy, and is insufficient as the only serological screening in this population.^[51,52]^ On the other hand, the HEp-2 IFA test may be useful, as some specific patterns suggest the presence of anti-ARS. The International Consensus on ANA Patterns has classified 30 clinically relevant HEp-2 IFA patterns with a respective alpha-numeric code.^[53]^ Several anti-ARS antibodies yield a cytoplasmic pattern, such as the cytoplasmic fine speckled pattern (AC-20) associated with anti-Jo-1, the cytoplasmic dense fine/quasi-homogeneous speckled pattern associated with anti-PL-7 or anti-PL-12, and the cytoplasmic variable-intensity fine speckled pattern associated with anti-MDA-5 antibodies. These patterns provide important information for integration with the results of autoantigen-specific immunoassays and the clinical presentation of patients (http://anapatterns.org, accessed July 20, 2023).^[54]^

### 4.4. Treatment

The treatment applied in this cohort was similar to that described in the literature, with different therapeutic options showing similar treatment responses. Pharmacological treatment is based on immunosuppression, which reduces the transcription of mediators that promote inflammation and the progression to fibrosis. Corticosteroids (0.5–1.0 mg/kg) must be used in association with immunosuppressants at the remission-inducing stage.^[29,55,56]^ For mild to moderate diseases, the first option is the use of corticosteroids with mycophenolate (sodium 360 or mofetil 500 mg: 03 capsule 12/12 h) or azathioprine (2.0–3.0 mg/kg/d). In cases of treatment failure or respiratory failure, rituximab (1 g, repeated in 2 weeks; course every 6 months) or cyclophosphamide (intravenous 0.5–0.75 g/m^2^ every 4 weeks) are used.^[57–59]^

If those fail, switching from 1 therapy to another or to another drug class, such as tacrolimus (0.1–0.2 mg/kg/d divided twice daily) or cyclosporine (4–6 mg/kg/d divided twice daily), is recommended.^[60,61]^ Antifibrotics should be considered in progressive cases.^[62]^ There is no preestablished duration for treatment, but similar to other systemic autoimmune diseases, it is usually not <2 years.^[63]^

### 4.5. Survival

Survival in the present cohort was better than that found in studies of progressive ILD in the literature.^[64]^ As in other studies, patients with the honeycombing pattern on HRCT had a poorer survival rate.^[65–67]^ Antifibrotics should be considered in these cases.^[68]^

In the current study, we found that a longitudinal reduction of ≥5% in the predicted FVC was associated with lower survival. To our knowledge, this is the first study on ILD with IIM showing that the decrease of ≥5% in the predicted FVC can be used to predict survival using spirometry.

### 4.6. Limitations

The main limitation of this study is retrospective nature, which can cause data loss despite recording data using a standardized form. In addition, the sample size was relatively small, as it is a rare disease. Furthermore, not all treated patients with mechanic’s hands underwent the extended antibody panel. It should be noted that the present sample included only outpatients with ILD; thus, these results should not be extended to cases with severe respiratory failure.

The absence of objective measurements of muscle strength, including respiratory muscle strength, was also a limitation, as well as the median value of muscle enzymes, which were described in the medical records as significantly elevated or not. Although the lungs are the most affected organ, it is noteworthy that there have been no studies on ILD and mechanic’s hands measuring respiratory muscle weakness. Moreover, we could not perform any analysis using D_L_CO due to the small number of patients who underwent this test, and no 6-minute walk test was performed. And finally the availability of antifibrotic medications to treat these patients, which continues to be a reality in many countries.

## 5. Conclusions

Outpatients with ILD associated with mechanic’s hands respond well to standard immunosuppressive treatment. Honeycombing on HRCT and a decrease in FVC ≥5% are predictors of lower survival.

## Author contributions

**Conceptualization:** Gustavo Frazatto Medeiros de Miranda, Maria Raquel Soares, Alexandre Wagner Silva de Souza, Luis Eduardo Coelho Andrade, Carlos Alberto de Castro Pereira.

**Data curation:** Gustavo Frazatto Medeiros de Miranda, Carlos Alberto de Castro Pereira.

**Investigation:** Gustavo Frazatto Medeiros de Miranda, Carlos Alberto de Castro Pereira.

**Methodology:** Gustavo Frazatto Medeiros de Miranda, Carlos Alberto de Castro Pereira.

**Project administration:** Gustavo Frazatto Medeiros de Miranda, Carlos Alberto de Castro Pereira.

**Writing—original draft:** Gustavo Frazatto Medeiros de Miranda, Carlos Alberto de Castro Pereira.

**Writing—review & editing:** Maria Raquel Soares, Alexandre Wagner Silva de Souza, Luis Eduardo Coelho Andrade, Carlos Alberto de Castro Pereira.

**Formal analysis:** Carlos Alberto de Castro Pereira.

**Supervision:** Carlos Alberto de Castro Pereira.

**Validation:** Carlos Alberto de Castro Pereira.

**Visualization:** Carlos Alberto de Castro Pereira.
